# Sequencing and analysis of the complete mitochondrial genome of *Changeondelphax velitchkovskyi* (Hemiptera: Fulgoroidea)

**DOI:** 10.1080/23802359.2017.1422407

**Published:** 2018-01-05

**Authors:** Yi-Xin Huang, Dao-Zheng Qin

**Affiliations:** Key Laboratory of Plant Protection Resources and Pest Management of the Ministry of Education Entomological Museum, Northwest A&F University, Yangling, Shaanxi, China

**Keywords:** Delphacidae, mtDNA, phylogenetic relationship

## Abstract

We determined the complete mitochondrial genome sequence of *Changeondelphax velitchkovskyi* (Melichar 1913). The complete mitogenome sequence of *C. velitchkovskyi* was observed to be a circular molecule 16,449 bp long and consists of 13 protein-coding genes (PCG), two ribosomal RNA (rRNA) genes and 22 transfer RNA (tRNA) genes (GenBank accession no. MG049916). This nucleotide composition is biased toward adenine and thymine (75.7% A + T). The A + T-rich region is found between *rrnS* and *trnI*, and this entire region was 1781 bp long.

*Changeondelphax velitchkovskyi* (Melichar 1913) is widely distributed in the Palaearctic Region. Unlike most delphacids that feed on Poaceae, it feeds on both Poaceae and Typhaceae (Kwon 1982; Ding 2006). However, the mitogenome sequence of *C. velitchkovskyi* remain unknown so far. Here, we sequenced the complete mitochondrial DNA genome of *C. velitchkovskyi* to provide more comprehensive data toward establishing its relationship within the family Delphacidae.

Adult *C. velitchkovskyi* males were collected from LONGFENG wetland in Daqing City (N 46°30′41.54″ and E 125°06′58.78″), Heilongjiang, China in August of 2014. Voucher specimens were deposited in the Key Laboratory of Plant Protection Resources and Pest Management of the Ministry of Education, Entomological Museum, Northwest A&F University (NWAFU).

The complete mitochondrial genome of *C. velitchkovskyi* was sequenced using an Illumina HiSeq2000 system made by the Shanghai Personal Biotechnology Limited Company (Shanghai, China). The annotation was carried out in Geneious 8.1.3 (Kearse et al. 2012). Protein-coding genes (PCG) were determined by the open reading frames; rRNAs and tRNAs were identified using MITOS (Bernt et al. 2013).

The *C. velitchkovskyi* mitochondrial genome is 16,449 bp (GenBank accession no. MG049916) in length with a total A + T content of 75.7% that is heavily biased toward the A and T nucleotides. It encodes the complete set of 37 genes which are usually found in animal mitogenomes. The gene arrangement in the mitochondrial genome of *C. velitchkovskyi* is conserved, similar to other mitogenomes in the Delphacidae, with the exception of *Nilaparvata lugens* (Zhang et al. 2013). In the mitogenome of *C. velitchkovskyi*, a total of 19 bp overlaps have been found at six gene junctions (*trnQ* and *trnM* share a nucleotide; *atp8* and *atp6* share seven nucleotides; *nad3* and *trnA* share two nucleotides; *trnN* and *trnS_1_* share one nucleotide; *trnS_1_*and *trnE* share one nucleotide, and *nad4* and *nad4L* share seven nucleotides). The mitogenome is loose and has a total of 486 bp intergenic sequences without the putative A + T-rich region. The intergenic sequences are at 15 locations ranging from 1 to 217 bp, with the longest one located between *trnS_2_* and *nad1*. The A + T-rich region of the *C. velitchkovskyi* is 1781 bp long and located between the *rrnS* and *trnI* genes.

All 22 tRNA genes usually found in the mitogenomes of insects are present in *C. velitchkovskyi*. The nucleotide length of tRNA genes ranges from 58 bp (*trnS_2_*) to 71 bp (*trnK*), and A + T content ranges from 66.7% (*trnI*) to 86.7% (*trnE*). These two rRNA genes have been identified on the N-strand in the *C. velitchkovskyi* mitogenome.

We analyzed the nucleotide sequences of 13 PCGs using the maximum likelihood (ML) method to understand the phylogenetic relationship of *C. velitchkovskyi* with other Auchenorrhyncha species. The mitogenome sequence of *Aphis gossypii* was used as the outgroup. Our results show that *C. velitchkovskyi* belongs to the superfamily Fulgoroidea and is clustered into a branch of Delphacidae (Figure 1). The family Delphacidae is monophyletic and *C. velitchkovskyi* is nested within the delphacids.

**Figure 1.**
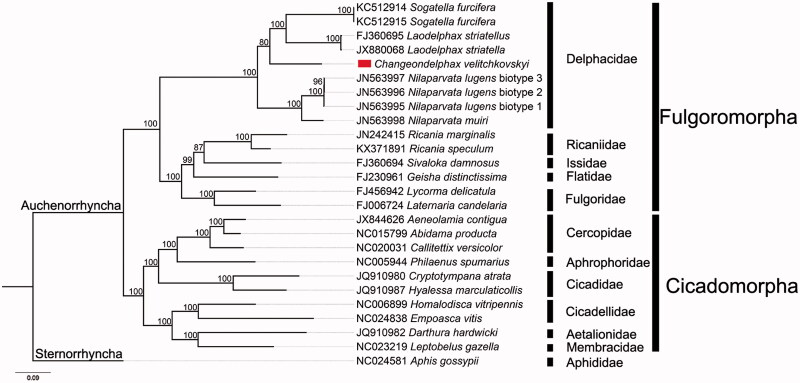
The maximum-likelihood (ML) phylogenetic tree of *C. velitchkovskyi* and other Auchenorrhyncha species. The numbers beside the nodes are percentages of 1000 bootstrap values. Alphanumeric terms indicate the GenBank accession numbers.
